# Changes in Early Cortical Visual Processing Predict Enhanced Reactivity in Deaf Individuals

**DOI:** 10.1371/journal.pone.0025607

**Published:** 2011-09-29

**Authors:** Davide Bottari, Anne Caclin, Marie-Hélène Giard, Francesco Pavani

**Affiliations:** 1 Department of Cognitive Sciences and Education, University of Trento, Trento, Italy; 2 Center for Mind/Brain Sciences, University of Trento, Trento, Italy; 3 INSERM, U1028; CNRS, UMR5292; Lyon Neuroscience Research Center, Brain Dynamics and Cognition Team, Lyon, France; 4 University Lyon 1, Lyon, France; French National Centre for Scientific Research, France

## Abstract

Individuals with profound deafness rely critically on vision to interact with their environment. Improvement of visual performance as a consequence of auditory deprivation is assumed to result from cross-modal changes occurring in late stages of visual processing. Here we measured reaction times and event-related potentials (ERPs) in profoundly deaf adults and hearing controls during a speeded visual detection task, to assess to what extent the enhanced reactivity of deaf individuals could reflect plastic changes in the early cortical processing of the stimulus. We found that deaf subjects were faster than hearing controls at detecting the visual targets, regardless of their location in the visual field (peripheral or peri-foveal). This behavioural facilitation was associated with ERP changes starting from the first detectable response in the striate cortex (C1 component) at about 80 ms after stimulus onset, and in the P1 complex (100–150 ms). In addition, we found that P1 peak amplitudes predicted the response times in deaf subjects, whereas in hearing individuals visual reactivity and ERP amplitudes correlated only at later stages of processing. These findings show that long-term auditory deprivation can profoundly alter visual processing from the earliest cortical stages. Furthermore, our results provide the first evidence of a co-variation between modified brain activity (cortical plasticity) and behavioural enhancement in this sensory-deprived population.

## Introduction

When coping with profound sensory deprivation, processing of inputs from the intact modalities is critical. In profound deafness, detection of changes in the environment and orienting of attention occurs primarily through vision. Deaf individuals are faster at detecting abrupt visual onsets [1], [2], [3], [4], faster and more accurate at discriminating motion direction of visual stimuli [5], and more efficient in re-orienting attentional resources in visual space [6], [7], [8]. These abilities have mainly, though not exclusively, been documented for events occurring towards the periphery of the visual field [9].

For profound deafness, it has been proposed that crossmodal changes involving the visual modality occur beyond the early processing stages, through the recruitment of higher-order visual areas as well as the de-afferented auditory cortices [10]. Differences in brain activation between deaf and hearing individuals have been found in the dorsal processing pathway (e.g., MT, MST) in response to motion stimuli [11], [12]. In addition, responses to visual stimuli have been recorded outside visually responsive brain regions, in the primary and secondary auditory cortices of deaf individuals [13], [14]. Recently Lomber and colleagues [15] showed in deaf cats a causal relationship between the reorganization of portions of the auditory cortex and specific enhancements of visual functions. Finally, two event-related potential (ERP) studies in humans [5], [16] found that the visual cortical responses elicited by deaf and hearing individuals started to differ from 180–200 ms latency, in the N1 component known to be generated in extra-striate cortex [17] and sensitive to high-order cognitive processes such as attentional orienting [18].

While ERPs are ideally suited to address the question of when changes in sensory processing occur, the possibility to reveal effects of crossmodal plasticity in early visual processing likely depends upon the specific task demands. The observation of modulations starting from the N1 component, for instance, could result from the use of visual discrimination tasks [5]. Indeed, target identification requires stimulus-feature binding before the response choice: this in turn necessitates orienting of attention towards the stimulus, which is known to modulate the N1 component of visual ERPs [18]. A simple detection task would not imply such processes and could reveal deafness-induced changes in earlier visual processing stages. In the present study, we tested this hypothesis by measuring reaction times and ERPs while deaf and hearing adults performed a simple speeded detection task. We focused our analyses on early (C1, P1) and later (N1) ERP components. In addition, we analysed the relationship between response times and visual ERP components, to highlight possible correlations between behavioural changes and brain responses in the deaf. As a large body of literature suggests that the deaf display enhanced visual performance particularly when the task involves the peripheral portion of the visual field [3], [5], we asked our participants to detect targets presented at either peri-foveal or peripheral eccentricities.

## Methods

### Participants

Ten profoundly deaf individuals (mean age = 33 years, SD = 4, range 18–50 year-old; mean years of education = 14, SD = 2.6; 6 female) were recruited at the National Association for Deaf (Ente Nazionale per la protezione e assistenza dei Sordi, Trento, Italy) and gave their informed consent to participate in the study. All deaf participants had uncorrected bilateral profound hearing loss (>90 dB), and acquired deafness within the first 3 years of age (8 had congenital deafness). All deaf participants were proficient sign-language users (6 learned sign language as first language, the other four had first a training based on Italian lip reading). Ten hearing matched controls (mean age = 29 years, SD = 2.5, range 23–50 years old; mean years of education = 16, SD = 2.6; 5 females) were also recruited to take part in the study. All participants had normal or corrected-to-normal vision and were right-handed by self-report.

The study was approved by the ethical committee at the University of Trento (Italy), and written informed consent was obtained from all participants prior to testing.

### Stimuli and apparatus

**Figure 1:**
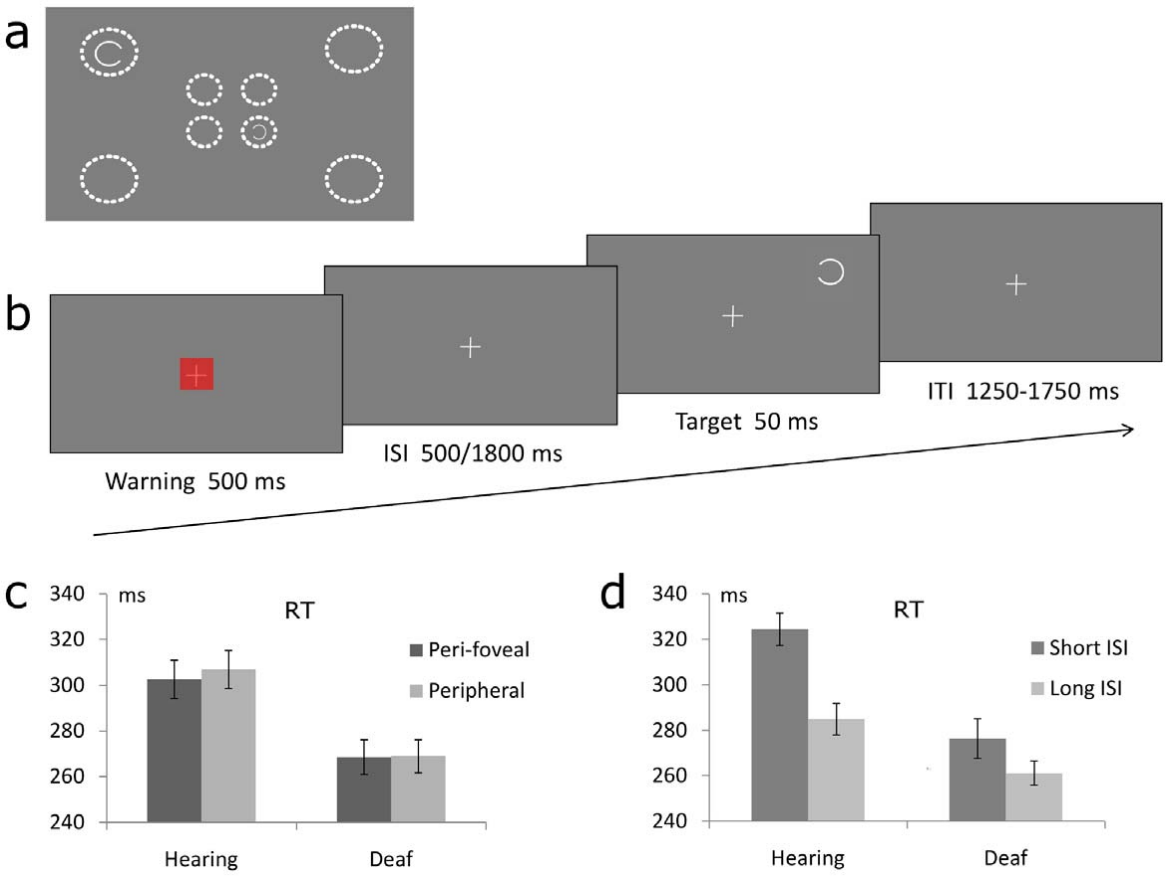
Experimental protocol and behavioural results. (**a**) The target was a single circle opened on the left or right side, presented at one of 8 possible locations. Peri-foveal targets were centred at 3° from fixation and covered a visual angle of 1.5°; peripheral targets were centred at 8° from fixation and covered a visual angle of 2.6° (i.e., targets were corrected for the cortical magnification factor). Dotted place-holders and examples of targets are shown in (a) for illustrative purposes only. (**b**) Each trial began with a warning signal (a red square covering 1.5° of visual angle, presented for 500 ms). The inter-stimulus intervals (ISIs) between the warning signal and the target were, equiprobably, either 500 ms (short ISI) or 1800 ms (long ISI). The target appeared for 50 ms, at any of the 8 possible locations randomly. Participants were instructed to press the response button as soon as possible. The inter-trial interval (ITI) ranged randomly between 1250 and 1750 ms. (**c, d**) Mean (of individual subjects medians) response times (RTs) for deaf and hearing participants as a function of (**c**) target eccentricity (perifoveal or peripheral) and (**d**) ISI (short or long) between the warning and the target. Deaf were overall faster than hearing controls. In addition, they showed no RT cost when reacting to peripheral vs. perifoveal targets, unlike hearing controls (**c**). Finally, the ISI modulated the reactivity performance in the two groups (**d**).

Visual fixation was a white cross, presented at the centre of the screen throughout the experimental session. The target was a circle opened on left or right side that could be presented at eight possible locations arranged on two invisible concentric circles centred on visual fixation. The radius of the inner circle was 3 degrees of visual angle, and the radius of the outer circle was 8 degrees. Four possible target locations were on the inner circle and four were on the outer (see Fig. 1a). Each location was placed along the two diagonals of the screen, thus resulting in 4 possible stimulus locations in the upper portion of the visual field, and 4 in the lower. From now on we will refer to locations on the inner circle as peri-foveal, and locations on the outer circle as peripheral. Targets appearing at peripheral locations were corrected for the cortical magnification factor [19]. Peri-foveal targets covered a visual angle of 1.5° and peripheral targets of 2.6°. The widths of the lines of the open circles were 1.5 mm and 2.4 mm for the peri-phoveal and peripheral targets, respectively. All stimuli were clearly presented suprathreshold. The luminance of the background was Y = 23, x = .278, y = .301, that of the target stimulus was Y = 83.3, x = .277, y = .297, and that of the cue was Y = 24.2, x = .510, y = .301 (measured with Chroma Meter CS-100A, http://www.konicaminolta.com/). All stimuli were presented on a standard 17 inches monitor, with 1024×768 pixels resolution, and 60 Hz refresh rate. The experiment was programmed with E-Studio 1.1.4.1, and run using E-Prime 1.1.4.1 (http://www.pstnet.com).

### Procedure

Participants sat at approximately 60 cm from the computer monitor inside a sound-attenuated chamber and were instructed to keep their head and eyes oriented towards fixation throughout testing. The experimental session lasted approximately 60 min. All hearing participants wore ear-plugs.

Each trial began with a warning stimulus consisting of a red square covering 1.5° of visual angle and presented for 500 ms (the visual fixation cross was superimposed on the red square, see Fig. 1b). The warning stimulus was presented for several reasons: first to give an attentional cue to the participants, second to analyze the ERPs to an event placed at fixation, and third to replicate our previous behavioural findings using the same paradigm [3]. The inter-stimulus intervals (ISIs) between the warning stimulus (offset) and the target (onset) were, randomly and equiprobably, either 500 ms (short ISI) or 1800 ms (long ISI). The visual target was presented randomly at any of the 8 possible locations (i.e., 4 central and 4 peripheral) for 50 ms. Participants were required to press as fast as possible the space bar of a computer keyboard upon detection of the target. The inter-trial interval (target offset to warning onset) ranged randomly from 1250 to 1750 ms. This task was designed on the basis of the paradigm used by Bottari and colleagues [3], which showed RT differences between deaf and hearing participants.

In case of anticipated responses (in the first 100 ms after stimulus onset), the sentence “do not anticipate!” appeared on the screen. Before data recording, participants completed a practice block of 24 trials. The experimental session was divided into 10 blocks comprising 96 trials each and lasting approximately 5 minutes. Between blocks, participants were invited to take short breaks. The experiment was a 2 by 2 by 2 factorial design, with target eccentricity (peri-foveal or peripheral) and ISI (short or long) as within-participant factors, and group (deaf individuals or hearing controls) as between-participants factor.

### Event-related potentials

The EEG was recorded (analog bandwidth: 0.1–200 Hz, sampling rate: 1 kHz) from 34 scalp sites using the International 10–20 System extended montage (documentation in http://www.easycap.de). Standard 10–20 sites were Fp1, Fp2, F7, F3, Fz, F4, F8, T7, C3, Cz, C4, T8, T5 (P7), P3, Pz, P4, T6 (P8), O1, and O2. Additional intermediate sites were FC5, FC1, FC2, FC6, TP9 (M1), CP5, CP1, CP2, TP10 (M2), PO3, PO4, PO9, Iz, and PO10. All scalp channels were referenced to the nose. Horizontal eye-movements were monitored with a bipolar montage from electrodes at left and right outer canthi. To eliminate the artefacts due to blinks, we performed an Independent Component Analysis (ICA; [20], [21]; runica version, implemented on EEGLAB running in MATLAB, http://www.mathworks.com/). The ICA was conducted on EEG epochs running from 1250 ms before the warning signal onset to 2200 ms after (for short-ISI trials), or 3500 ms after (for long-ISI trials). After removal of blink effects, trials including incorrect responses (anticipations, i.e. responses before the target or in the 100 ms following it) or with signals exceeding 100 µV at any electrode were excluded from averaging.

ERPs to the warning signal were averaged over a period of 2700 ms including 500 ms pre-stimulus (on average, 680 trials after artefact rejection). ERPs to the targets were averaged separately for each ISI and target location over a period of 1000 ms including 500 ms pre-stimulus (on average, 188 trials per condition). The responses were corrected relative to a [−100, 0 ms] pre-stimulus baseline with respect to both warning signal and target onsets. In addition, the ERPs to targets were also corrected relative to a [−300, −200 ms] baseline to assess possible group differences in anticipatory activities occurring before the target onset and their potential effects on the subsequent brain responses. Finally, the ERPs were digitally filtered (low-pass filter with a 30-Hz cut-off, slope 24db/octave). Scalp potential maps were generated using spherical spline interpolation [22], [23]. All EEG data were analysed with the ELAN-pack software developed at the Brain Dynamics and Cognition team in Lyon [24].

### Statistical approach

Before comparing the cortical activity between groups, we evaluated the emergence of visual evoked responses within each group. To this purpose, we run a series of Wilcoxon tests across all participants, comparing to zero the activity recorded at each time sample. We performed this exploratory analysis for the ERPs to the warning signal and to the targets, to select the time windows and electrodes for the main analyses. The selected time windows were thus: C1 [40–95 ms], P1 [80–150 ms] and N1 [150–220 ms]. Main analyses were conducted on peak amplitude and peak latency for the C1, P1 and N1 components, adopting mixed ANOVAs and between-group t-tests. When necessary the Tukey test was used for post-hoc comparisons.

## Results

### Enhanced visual reactivity in deaf relative to hearing individuals

Response times (RTs) in deaf and hearing participants were computed as the medians for each participant in each condition, and analyzed using an ANOVA with Target Eccentricity (3° or 8°) and Inter-stimulus interval (ISI) between warning signal and target (short or long) as within-subject factors, and Group (deaf, hearing) as between-subject factor. Deaf participants were overall faster than hearing controls at detecting the visual targets (269±10 (SEM) ms vs. 305±10 (SEM) ms, respectively; F(1,18) = 6.7, p<0.02). In addition, while hearing controls showed a small but consistent RT cost (4 ms; t(9) = 2.6, p<0.03) when responding to peripheral relative to peri-foveal targets, this cost was negligible in deaf participants (0.4 ms; t<1). This resulted in a marginally significant interaction between Group and Target Eccentricity (F(1,18) = 3.3, p<0.08; see Fig. 1c). Importantly, the faster RTs found in the deaf group compared to hearing controls was not due to a larger number of anticipation responses, as the amount of trials in which the RTs were below 100 ms was comparable in the two groups (proportion of anticipation trials: Hearing = 0.0004±0.0002; Deaf = 0.0009±0.0004; t(18) = 1.2, p = 0.2). Overall, these findings replicate previous reports [1], [2], [3] and indicate that profound deafness can enhance reactivity to visual events, particularly towards the periphery of the visual field. An additional behavioural finding was the significant interaction between the warning-to-target ISI and Group factors (F(1,18) = 8.8, p<0.01; see Fig. 1d). Hearing controls were faster at detecting targets at long than short ISI (285±10 ms vs. 324±10 ms, respectively; p<0.0002, Tukey HSD post-hoc test), as predicted by the increased posterior probability for the long ISI targets [25]. This ISI-related difference did not significantly emerge in deaf individuals (261±8 ms vs. 276±12 ms). As a result, reactivity difference between groups was substantial at the short ISI (p = 0.02), and not significant at the long ISI (p = 0.4, Tukey HSD tests). Note however that a ceiling effect in deaf individuals might explain this last result.

### Brain responses to the central warning signal

The warning signal elicited “classical” visual ERP components on posterior scalp areas in both groups, with however differences between deaf and hearing participants in the latency, amplitude, and morphology of the early visual responses (C1, P1, N1 components).

**Figure 2:**
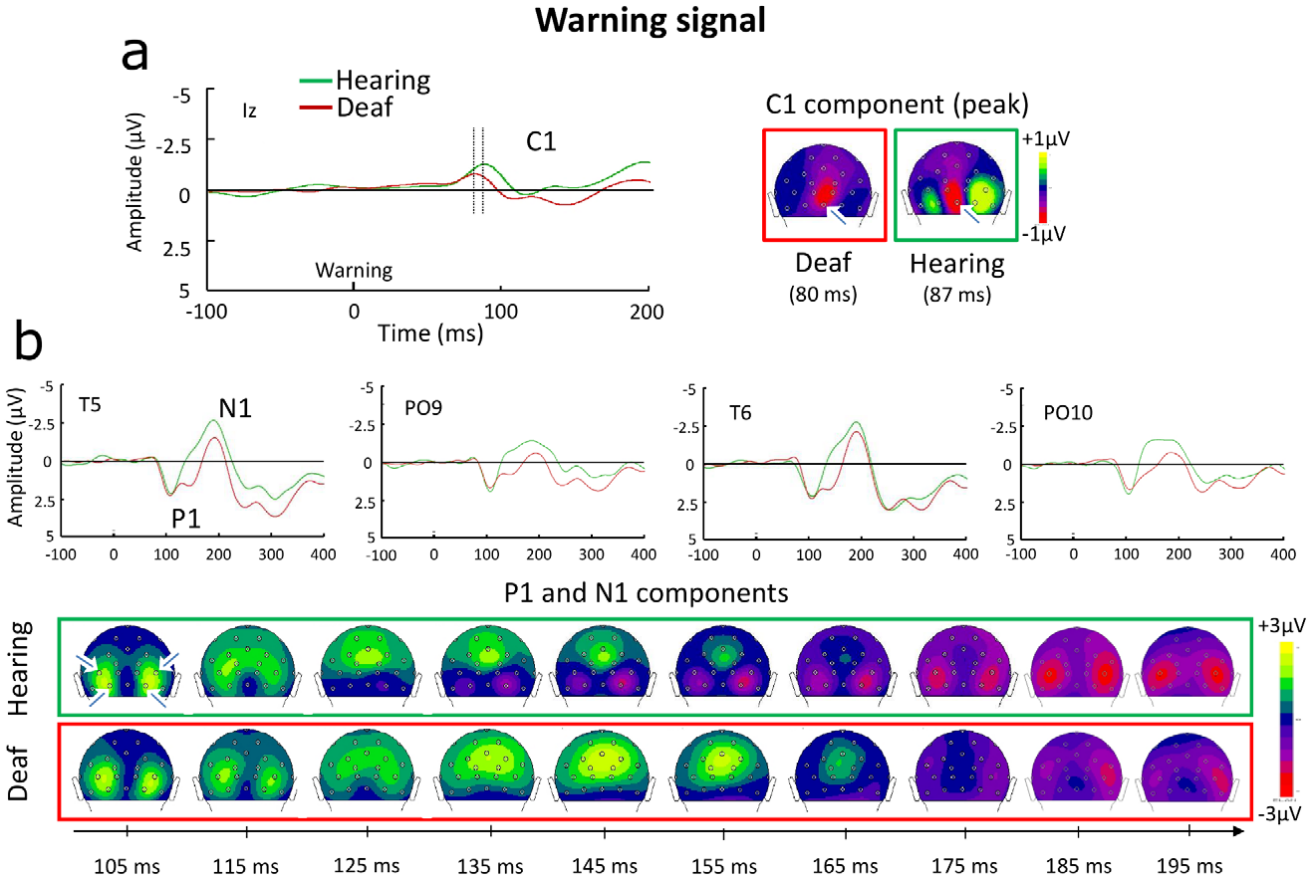
Brain responses to the central warning signal in deaf subjects and in hearing controls. (**a**) Visual ERPs and topography of the C1 component around its peak latency for each group. Arrows indicate the electrode (Iz) at which the ERP curves are shown. (Note that because the C1 peak latency is later in hearing controls than in deaf participants, the emerging P1 can be seen for the controls on each side of the C1 in this figure.) (**b**) P1 and N1 responses at 4 posterior electrodes (T5, PO9, T6, PO10, identified by arrows in the first potential map). The P1 components have a similar profile in the two subjects groups until about 125 ms, then deaf subjects present a second positive deflection (145 ms) compared to hearing controls. The prolonged positivity in deaf subjects (145–165 ms) is clearly seen in the spatiotemporal distribution of the responses (back view of the head). By contrast, although the negative N1 component emerged earlier in control than in deaf subjects, there was no difference in morphology or topography between the two subjects groups.

Both deaf and hearing participants exhibited a significant C1 component of negative polarity [26] within the 40–95 ms time interval, with a maximum amplitude around Iz electrode (see Fig. 2a). Its peak latency was significantly shorter in deaf individuals (80±2 ms) than in hearing participants (87±2 ms; t(18) = 2.3, p<0.04). There was no significant group difference in peak amplitudes (t(18)<1.4). This difference in the C1 peak latency between groups would indicate that the cortical processing between deaf and hearing subjects already differs at the level of the striate cortex [17]. An increasing number of evidence suggests that visual attention might modulate the C1 component [27], [28], [29], [30]. Nonetheless all the observed C1 modulations as a function of attentional and/or perceptual load manipulations emerged as amplitude changes. The present latency difference might reflect changes in striate cortex activity or even in earlier processing stages of deaf individuals' visual pathway. In any case, this result shows that differences in visual processing between deaf individuals and hearing controls can emerge for a central visual event, presented directly at fixation.

Following the C1 deflection, a prominent P1 component was recorded over the posterior electrodes between 80 and 150 ms, with a substantially different morphology between the two groups. As shown in Fig. 2b, the two groups present a similar P1 profile until about 125 ms, with a positive peak around 105 ms. Then, unlike hearing controls, deaf displayed a second positive deflection around 145 ms. This double deflection was observed in 7 out of 10 deaf participants (but only in 2 out of 10 hearing controls). To assess the consistency of these different P1 profiles, we compared between the two groups the mean amplitude of the P1 response in two different latency ranges: 105 ms±10 ms (first P1 peak), and 145 ms±10 ms (second P1 deflection), over the subset of posterior electrodes that better captured this activity (T5-T6-PO9-PO10-TP9-TP10). As expected from the ERP waves in Fig. 2b, the mean amplitude around the first P1 peak did not differ between the two subjects' groups (t(18)<0.4), whereas around the second P1 peak deaf individuals displayed a greater positive activity (0.9±0.6 µV) compared to hearing controls (−1.2±0.8 µV , t(18) = 4.1, p<0.05). The different morphology of the P1 complex between groups is also evident in the spatiotemporal distribution of the responses between 105 and 195 ms (see lower panel in Fig. 2b). From about 135 to 165 ms, while hearing controls present negative potential fields at the most occipital electrodes (emerging N1 component), deaf individuals still show an enduring positive activity centred over parietal electrodes. This corroborates further our findings that cortical changes in deaf compared to hearing individuals can be detected in early processing and for a centrally displayed stimulus (see also [31] for a result in this direction, showing ERP differences at the P2 level, between cochlear implanted patients and hearing controls for visual stimuli presented at fixation).

Although visual inspection of the topographies (see Fig. 2b) suggests that the N1 component emerged earlier in controls than in deaf individuals around the occipital electrodes (e.g. PO9 and PO10), its morphology was similar between the two groups, and there was no significant difference in the peak amplitude (5.3 vs. 5.0 µV) or peak latency (177 vs. 180 ms, hearing controls vs. deaf individuals respectively, both ts<1).

### Brain responses to peri-foveal and peripheral targets

**Figure 3:**
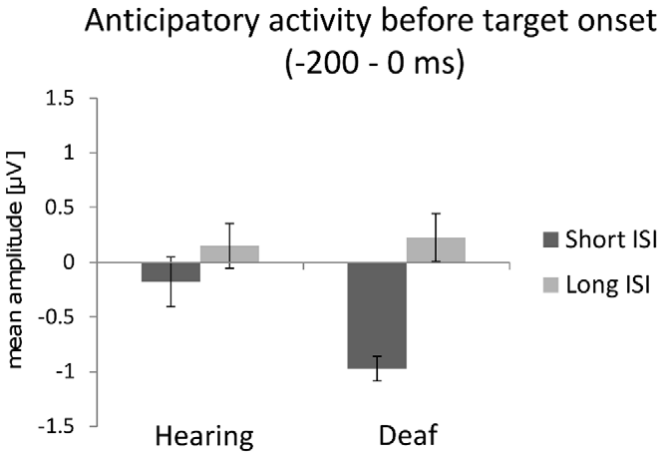
Anticipatory activity recorded before target onset. Deaf individuals show enhanced anticipatory activity (mean amplitude over the 200 ms preceding target onset, with a −300 to −200 ms baseline) compared to hearing controls for target presented at short ISI.

In paradigms using a warning signal prior to the occurrence of a visual target, subjects may generate anticipatory activities in sensory cortices before target appearance. To test for this possibility, we measured in each subject the mean ERP amplitude over the 200 ms preceding the target (relative to a baseline taken over the [−300, −200 ms] pre-stimulus period), averaged across parieto-occipital electrodes (PO3-PO4-O1-O2-PO9-PO10-Iz). This mean pre-stimulus activity did not differ from the baseline for either (short or long) ISI condition in the control group. It was however significantly different from the baseline in the deaf group for the targets presented at short (but not at long) ISI (t(9) = 4.5, p<0.005). This result was further confirmed when comparing the mean pre-stimulus amplitudes of the two groups in a two-way ANOVA with the factors ISI and Group, with a significant interaction between the two factors (F(1,18) = 4.6, p<0.05): this was caused by greater mean amplitude (sustained anticipatory activity) in deaf than hearing participants when the target was presented at short ISI (p = 0.05, Tukey HSD post-hoc test; Fig. 3). Because the different anticipatory activities in deaf and hearing subjects (over the 200 ms preceding the target presentation) could impact differently on subsequent brain responses, we kept the same [−300, −200 ms] pre-stimulus baseline for ERP component analyses. For completeness, however, we also report the results of the analyses when adopting a [−100, 0 ms] baseline, as for the warning signal, in Table 1. The results remain globally unchanged irrespective of the adopted baseline.

**Table 1:** Analyses (ANOVAs) of the ERPs to targets and their relationships with behavioural responses relative to a baseline taken over the 100 ms preceding the target onset.

**Brain responses to peri-foveal and peripheral targets**			
P1 component	Factors	- 2 within-subjects: Target Eccentricity (peri-foveal, peripheral), ISI (short, long) - 1 between-subjects: Group	
	Peak latency	*Main effects*	ISI: F(1,18) = 8.7, p<0.01Target Eccentricity: F(1,18) = 6.5, p<0.02**Group**: F(1,18) = 5.7, p<0.03
		*No interactions involving Group*	All Fs<1
	Peak Amplitude	*Main effects*	ISI: F(1,18) = 5.7, p<0.03
		*Interactions involving Group*	**ISI×Group**: F(1,18) = 13.4, p<0.01
N1 component	Factors	- 2 within-subjects: Target Eccentricity (peri-foveal, peripheral), ISI (short, long) - 1 between-subjects: Group	
	Peak latency	*No main effects or interactions involving Group*	Target eccentricity: F(1,18) = 3.7, p<0.08
	Peak Amplitude	*No main effects or interactions involving Group*	All Fs<2.8
**Changes in early visual ERPs of the profoundly deaf predict enhanced reactivity**			
P1 component	Factors	- 2 within-subjects: ISI (short, long), Quartile (1,2,3,4)- 1 between-subjects: Group	
	Peak latency	*No main effects or interactions involving Group*	All Fs<1.8, n.s.
	Peak Amplitude	*Main effects*	ISI: F(1,18) = 8.01, p<0.02Quartile: F(3,54) = 6.3, p<0.001
		***Interactions involving Group***	ISI×Group: F(1,18)<1, n.s.**Quartile×Group**: F(3,54) = 3.5, p<0.02
		***Post-hoc*** ** ANOVAs ** ***for each group with ISI and Quartile as factors***	**Deaf: Quartile** F(3,27) = 8.3, p<0.001 (linear contrast F(1,9) = 15.7, p<0.003)Hearing: Quartile F<1.5, n.s. (linear contrast: F<1, n.s.)
N1 component	Factors	- 2 within-subjects: ISI (short, long), Quartile (1,2,3,4)- 1 between-subjects: Group	
	Peak latency	*No main effects or interactions involving Group*	All Fs<2.4, n.s.
	Peak Amplitude	*Main effects*	Quartile: F(3,54) = 14.7, p<0.0001 (linear contrast F(1,18) = 22.1, p<0.0001)
		*No Interactions involving Group*	All Fs<1

Significant effects involving the group factor are highlighted with a grey background. Note that the results are similar to those obtained with a [−300, −200 ms] baseline reported in the main text.

The C1 component elicited by the targets was observable in the grand average responses, with the typical inversion of polarity for upper vs. lower visual field stimulation [26]. However, it could not be further analyzed because it was not well defined in every participant.

**Figure 4:**
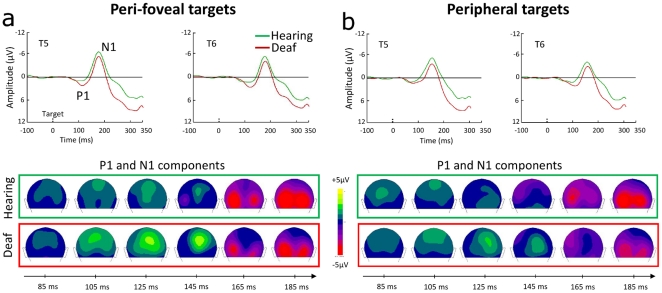
Brain responses to (a) peri-foveal and (b) peripheral targets. Upper panels show ERPs at T5 and T6 electrodes for the targets. Lower panels show spatiotemporal distribution of the responses between 85 and 185 ms in each group. Like in the responses to the warning signal, the P1 component is prolonged in deaf compared to hearing subjects, whereas the N1 is similar.

Visual inspection of the P1 component to targets did not reveal the clear biphasic morphology seen in the response to the warning signal (see ERP waves in Fig. 4). This can be due to the fact that P1 is usually sensitive to the visual contrast of the stimulus: this contrast was much lower for the targets than for the warning signal (red square). In addition, ERPs to the targets (peri-foveal or peripheral) are averaged responses to stimuli presented at four distinct locations (upper and lower, right and left positions, see Fig. 1a) while the warning signal was always delivered at fixation. Nevertheless, the spatiotemporal distribution of the responses to targets (see topographies in Fig. 4a and Fig. 4b) shows that the development of the P1 and N1 components in the two subjects' groups is similar to that observed in response to the warning signal: deaf display a prolonged P1 relative to hearing controls, with a prominent late phase distributed over parietal scalp sites.

The peak latencies and amplitudes of P1 (determined within the 80–150 ms time window across the posterior electrodes T5-T6-PO3-PO4-P3-P4-Pz-O1-O2-TP9-TP10-PO9-PO10-Iz) were compared between the two groups using ANOVAs with Target Eccentricity (peri-foveal or peripheral) and ISI (short or long) as within-participant factors and Group as between-participant factor. Analysis on P1 peak latency revealed a significant effect of ISI (F(1,18) = 8.4, p<0.01) due to a delayed P1 peak for targets presented at long compared to short ISI, and a marginally significant effect of Target Eccentricity (F(1,18) = 4.2, p<0.06), showing a tendency for delayed P1 peak for peripheral relative to central targets. Most importantly, deaf participants showed a significant peak latency delay (130±5 ms) compared to hearing controls (115±5 ms, F(1,18) = 4.8, p<0.05). There were no other significant interactions involving the Group factor (Fs<1.5). In particular, the absence of an ISI-by-Group interaction (F<1 with both baseline corrections) suggested that the difference between the two groups in anticipatory activities as a function of ISI reported above had no effect on the P1 peak latency. ANOVA on the P1 peak amplitude showed again a significant effect of ISI (F(1,18) = 13.3, p<0.01), indicating that participants generated larger P1 amplitudes to targets presented at long compared to short ISIs. The interaction between the ISI and Group factors tended towards significance (F(1,18) = 4.2, p<0.06). In particular, deaf tended to show a larger difference between the P1 peak amplitude in the long- compared to the short-ISI condition than hearing controls. (Note that this interaction may have contributed to the main effect of ISI reported above). Lastly, although it did not reach statistical significance, there was a trend for a larger P1 amplitude in deaf (4.6±0.6 µV) than in hearing subjects (3.2±0.6 µV, F(1,18) = 2.5, p = 1.3).

Analysis of the N1 component (assessed over the 150–220 ms time window) using the same procedure as for the P1 response did not reveal any significant effect of ISI or Group, nor interaction involving the Group factor (all Fs<3.5).

### Changes in early visual ERPs of the profoundly deaf predict enhanced reactivity

**Figure 5:**
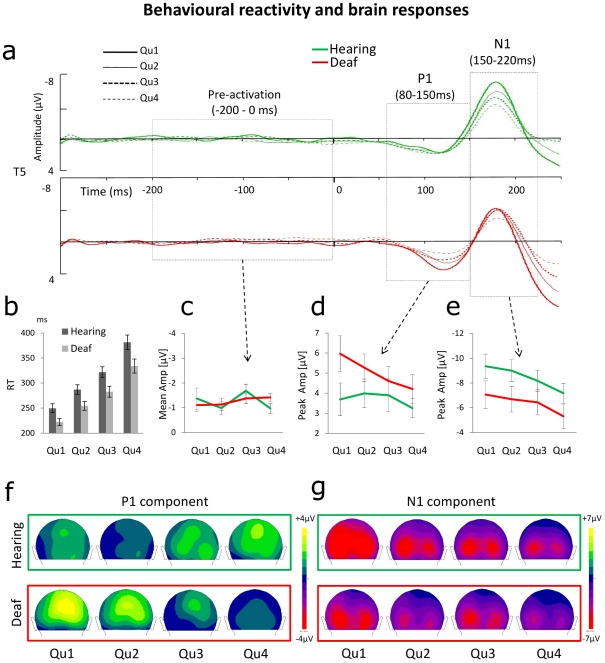
Correlation between behavioural reactivity and brain responses. The figure depicts the main ERP components as a function of RTs in deaf individuals and in hearing controls. (**a**, **b**) For each participant trials were sorted into 4 quartiles as a function of the response speed, from the fastest (Qu1) to the slowest (Qu4), and ERPs were averaged within each quartile. (**c**) In both groups the mean amplitude of the pre-stimulus activity (leftmost dashed area in (**a**) see text) was unrelated to the RTs. (**d**) By contrast, the peak amplitude of the P1 component (central dashed area in (**a**)) decreases linearly as a function of RTs in deaf participants, but not in hearing controls. (**e**) Finally, in both groups the peak amplitude of the N1 component (rightmost dashed area in (**a**) decreased monotonically as a function of RTs. The lower panel illustrates these results by showing the topographies of the P1 (**f**) and N1 (**g**) components around their respective peak latencies in both subjects' groups as a function of the four quartiles: the potential fields related to P1 peak decrease with increasing RTs only for the deaf group, whereas N1 potentials decrease similarly in the two groups with increasing RTs.

Having demonstrated substantial early visual ERPs differences between deaf and hearing subjects in particular in the P1 latency range, we turned to investigate whether these different ERP profiles could predict the observed behavioural differences in terms of reactivity to the visual targets. To this aim, we separated the RT distribution for each condition (target eccentricity and ISI) and in each participant into four quartiles (Fig. 5b). Trials belonging to each quartile were used to calculate four ERPs for each participant. We could thus re-analyze the main responses to the targets (anticipatory activities, P1, and N1 components) as a function of the RT quartiles. The overall outcome of these analyses is illustrated in Fig. 5.

As we reported above, deaf individuals showed enhanced sustained activity prior to the appearance of the target in the short ISI condition. Because we observed the larger behavioural advantage for deaf compared to hearing subjects precisely for the short ISI, we examined whether enhanced pre-stimulus activity (within the 200 ms prior to target presentation, relative to a [−300, −200 ms] baseline; see Figure 3) could be related to the RTs. A mixed ANOVA with ISI (short or long), Quartile (1, 2, 3, 4) and Group as factors did not reveal significant main effects of Group or Quartile (F(1,18) = 3, p<0.1 and F<1, respectively) on pre-stimulus activity, nor significant interactions between Quartile and Group, or between ISI, Quartile and Group (Fs<1; as expected from the previous analysis on the anticipatory activity, the ISI×Group interaction was significant F(1,18) = 4.4, p = 0.05). These results thus indicate that, whatever the ISI the RTs did not – at least directly – depend on anticipatory activities in visual areas before target appearance (Fig. 5c).

A very different pattern of results emerges when considering the peak amplitude of the P1 component as a function of ISI, Quartile, and Group (note that for this analysis data were pooled across target locations as this factor did not affect P1 or N1 latencies or amplitudes in previous analysis). An ANOVA with ISI, Quartile, and Group as factors revealed a significant main effect of ISI (F(1,18) = 13.7, p<0.01) and an interaction between ISI and Group (F(1,18) = 4.9, p<0.04) showing that deaf individuals displayed an enhanced P1 peak amplitude for targets presented at long than at short ISI (p<0.01, Tukey HSD post-hoc test). Most importantly, the analysis revealed a significant main effect of Quartile (F(3,54) = 5.1, p<0.01) and a significant interaction between Quartile and Group (F(3,54) = 3.7, p<0.02). A separate ANOVA on the deaf data showed a significant effect of Quartile (F(3,27) = 7.9, p<0.001), and a significant linear contrast (F(1,9) = 19.5, p<0.003; see Fig. 5d). Conversely, this relationship between ERP and behaviour was entirely absent in hearing controls (for both baseline corrections, main effect of Quartile: F<1.5; linear contrast: F<1).

In sum, RTs of deaf individuals slow down as P1 peak amplitude decreases, with a clear linear relationship, whereas no such relationship emerged in hearing controls. Figure 5f illustrates these effects in the topography of the P1 component (around its peak latency) as a function of quartiles. The same analysis on peak latency did not show any significant effect of Quartile (F<1 for both baseline corrections) nor any interaction involving the Group factor (F<1.8, n.s. for both baseline corrections).

Finally, in both deaf and hearing participants the amplitude of the N1 component predicted RTs. A similar relationship between ERPs and behaviour emerged in the two groups (Fig. 5e; see also Fig. 5g for the topography of the N1 peak as a function of quartiles). ANOVA on the N1 peak amplitude showed a significant main effect of Quartile (F(3,54) = 10.7, p<0.0001) and a linear pattern for this factor (linear contrast: F(1,18) = 17.5, p<0.001). However, no significant interaction involving the Group factor was found (F<1 for both baseline corrections). Thus, for both groups faster RTs were associated with an increase in N1 peak amplitude. Similar to the P1 component, there was no effect of the Quartile or Group factors on the N1 peak latency (F<2.4, n.s. for both baseline corrections).

## Discussion

The current view on visual changes in profound deafness is that they entail crossmodal plasticity resulting in modifications in late visual processing [9], [10]. Using a simple speeded detection task, our study challenges this dominant view by providing evidence for modulations of the brain responses in deaf individuals at very early visual processing stages. Neural responses to visual events differed between deaf and hearing participants already in the C1 ERP component (50–95 ms) associated with striate cortex activity. Furthermore, substantial differences between the two subjects' groups emerged in the latency range of the P1 component (80–150 ms) for both warning and target stimuli. The P1 complex was prolonged in deaf relative to hearing participants, with a biphasic morphology in 7 out of 10 deaf participants at least in the responses to the (salient) warning signal. This finding thus sets the initial hallmark for a divergence in visual processing between deaf and hearing individuals at the first cortical stages, about 100 ms before that documented in previous reports [5], [16].

Our findings also provide the first evidence that this altered dynamics of early visual ERPs may play a functional role in the changes observed at the behavioural level in the deaf. In both subjects' groups, the speed of the upcoming response was linearly related to the N1 peak amplitude. This finding corroborates previous evidence in hearing individuals [32] showing a linear relationship between RTs and N1 amplitude. Strikingly, our results also revealed that the relationship between the electrical brain response and reaction time dissociates between the two groups, precisely in the early ERP component that differs the most substantially between deaf and hearing individuals (i.e., the P1 complex). In the deaf group only, RTs decreased linearly with P1 peak amplitude . This calls for two remarks. First, it may seem paradoxical that RTs in the Deaf were not related to P1 latency (rather than amplitude) as this component was prolonged in this group, resulting in a change in its peak latency. As noted above however, this prolonged P1 complex stems more from a change in P1 morphology rather than an actual delay of the response. Second, the important point is that the ERP component that predicts the behavioural measures is anticipated in deaf (P1) compared to hearing subjects (N1) by a time globally corresponding to the anticipation of the behavioural responses (it is remarkable to note that the difference between the P1 and N1 peak latencies is around 50 ms, roughly the amount of difference between the RTs of deaf and hearing subjects).

To sum up, changes in initial stages of visual processing predict enhanced visual reactivity in deaf individuals. Thus, the reactivity advantage in this sensory deprived population cannot be ascribed to response preparation alone, but emerge also within the visual system before response release. This is also corroborated by two observations: (i) in behavioural measurements, the occasional anticipatory responses were comparable between groups, and (ii) in ERPs, the amplitude of preparatory activities did not predict RT advantages (Fig. 5c).

One possible account for the early visual changes we observed in deaf adults is linked to selective attention. Behavioural and neural changes in the visual processing of the profoundly deaf have been linked to changes in selective visual attention [5], [6], [9]. Although in our task orienting of attentional resources to the target was not a mandatory stage before the response (unlike in visual discrimination tasks), the visual onsets of the salient warning signal and the targets surely captured the participant's exogenous attention. The changes in the P1 dynamics in the deaf may thus reflect stronger exogenous attention capture in deaf compared to hearing subjects. This is compatible with recent data showing that exogenous attention can indeed affect the P1 ERP complex [33], and that specifically the second phase of the P1 component could be modulated by attentional capture [34].

Nonetheless, this attentional account cannot easily explain the latency difference observed in the C1 component. Although an increasing number of evidences suggests that attention and perceptual load can modulate the C1 component [27], [28], [29], [30], all the effects reported were expressed by C1 amplitude changes. As the C1 component reflects the initial stage of striate cortex activity, the observed latency difference could represent a plastic change in V1 or in an earlier visual stage in deaf individuals. As a general remark, it should be noted that morphological changes at various stages of the central nervous system as a consequence of profound deafness could indeed have contributed to the early changes we observed in visual ERPs. Recently Codina and colleagues (2011) [35] found evidence for cross-modal plasticity effects as early as at the retinal level (larger neural rim areas within the neural optic nerve head, suggesting a greater number of retinal ganglion cells in deaf individuals compared to hearing controls). In addition, at the subcortical level, aberrant retinal projections to the auditory thalamus and to the intermediate layers of the superior colliculus have been described in deaf mice [36]. Although to our knowledge, gray matter changes have not been documented in the visual cortex of deaf humans [14], the existence of atypical white matter fibers has been reported in deaf animals and deaf humans. At the cortical level, recent studies using DTI (diffusion tensor imaging) have reported increased anisotropy in the forceps major of the corpus callosum in early deaf adults, suggesting increased connectivity between visual cortices in these individuals [37], [38]. These morphological changes, observed at several stages of the visual pathway could be responsible for the modified dynamics of the C1 (striate cortex) and P1 (extra-striate cortices) ERP components in deaf compared to hearing subjects, without claiming an attentional explanation.

A final important aspect of our findings is that the ERP differences between deaf and hearing subjects were largely comparable for visual events at 3 or 8 degrees of eccentricity (targets) and, perhaps more surprisingly, even for visual events delivered at fixation (warning signal). This result is compatible with the observation of enhanced visual reactivity regardless of stimulus eccentricity in deaf compared to hearing subjects [4], but it contrasts with the widespread assumption that compensatory changes of visual processing in the deaf should emerge selectively for events appearing towards the periphery of the visual field. While a special role of the visual periphery in profound deafness is undisputed, and documented also by some aspects of the present findings (see behavioural results in Fig. 1c), our results clearly indicate that early changes in visual processing occur for events at both central and peripheral portions of the visual field.

In conclusion, the present findings extend previous views on the cross-modal effects of deafness on visual processing in various ways. First, they show that auditory deprivation can alter visual processing from the earliest cortical stages. Second, they reveal a link between reactivity to visual events in profound deafness and changes occuring at early stages of visual processing, thus providing the first evidence of co-variation between modified brain activity (cortical plasticity) and behavioural enhancement in this sensory deprived population. This suggests that the stage of visual processing which accumulates the critical perceptual evidence to trigger the simple detection response may be anticipated in deaf compared to hearing individuals. While speculative this interpretation of the relation between brain response and behavioural reactivity suggests that profound deafness may not just re-structure the spatial processing of the visual scene (with enhanced abilities for the periphery of the visual field), but may also modify the timing of visual functions.
